# Supplementary material for: Live Drosophila melanogaster Larvae Deter Oviposition by Drosophila suzukii
Source: Insects. 2022 Jul 29;13(8):688. doi: 10.3390/insects13080688 (PMC9408982; doi:10.3390/insects13080688)
Supplement: Supplementary file 1 [file insects-13-00688-s001.zip › Table S1.pdf]

Table S1. Compounds identified in hexane washes of adult *Drosophila melanogaster* (Dm) and *D. suzukii* (Ds)and their relative amounts (% composition). RT is retention time and RI is retention index relative to retention times of *n*-alkanes on a polar GC column. See main text for details of compound abbreviations.

			Relative amount (%)			
RT (min)	RI	Compound	Dm fem	Dm mal	Ds fem	Ds mal
15.89	2099	21H	1.45	2.25	10.61	10.28
16.80	2197	22H	0.70	1.06	0.71	0.63
17.00	2218	unsat HC	0.25	0.50	0.54	0.44
17.27	2247	2Me-22H	0.51	0.43	1.20	0.64
17.69	2299	23H	6.51	6.88	7.36	4.26
17.81	2313	Z9-23H	3.28	4.39	10.67	8.93
17.89	2323	Z7-23H	22.88	36.99	34.58	26.08
17.99	2334	Z5-23H	1.10	1.74	0.00	0.37
18.05	2341	ZZ7,11-23H	1.48	0.17	0.00	0.00
18.19	2357	ZZ6,9-23H	0.24	0.31	4.48	4.26
18.97	2452	2Me-24H	2.72	2.98	0.48	0.41
19.34	2499	25H	1.97	1.06	1.43	1.08
19.44	2511	unsat HC?	0.00	0.00	0.33	0.00
19.48	2516	Z9-25H	3.91	1.86	0.80	0.88
19.56	2527	Z7-25H	4.53	5.14	2.20	1.91
19.64	2537	ZZ7,11-25H	0.80	0.00	0.00	0.00
19.70	2544	Z11-18Ac(cVA)	5.74	12.60	0.00	0.00
19.85	2563	ZZ6,9-25H	0.65	0.35	1.48	1.34
20.46	2643	unsat HC	0.27	0.36	0.00	0.42
20.54	2653	2Me-26H	9.38	7.87	1.39	2.34
20.90	2701	27H	0.00	0.00	1.36	1.11
21.03	2719	Z9-27H	0.00	0.00	0.40	1.32
21.12	2731	Z7-27H	0.00	0.00	0.98	2.06
21.06	2723	20Ac	0.00	0.00	0.00	0.00
21.28	2752	ZZ7,11-27H	16.21	3.92	0.00	0.00
21.39	2767	ZZ6,9-27H	0.76	0.45	0.29	0.78
22.01	2849	2Me-28H	8.48	6.32	6.86	12.01
22.37	2897	29H	0.00	0.00	1.43	2.01
22.51	2916	Z9-29H	0.00	0.00	5.45	7.98
22.57	2924	Z7-29H	0.00	0.00	1.49	1.73
22.65	2935	Z5-29H	0.00	0.00	2.15	5.14
22.73	2945	unsat HC	0.00	0.00	0.52	0.00
22.84	2960	ZZ7,11-29H	4.12	1.05	0.00	0.00
23.77	3094	31H	2.06	1.30	0.82	1.58
